# Bibliographical

**Published:** 1883-08

**Authors:** 


### Bibliographical.

### OUR BOOK TABLE.

Journals there are in abundance devoted to some particular field of scientific enquiry, but until the issue of "SCIENCE," America could not boast of an illustrated weekly publication wholly set apart to the advancement of general scientific research. During the six months of its publication it has secured a standing and won a reputation of which older journals might well be proud, and it now occupies an established position as an authority upon all purely scientific questions. Our own Popular Science department has so often been under obligation to it for some of its most valuable items, that we should scarce know what to do were we deprived of its regular visits. It is published at Cambridge, Mass., at $5.00 per annum.

THE NEW YORK MEDICAL JOURNAL, published by the well known firm of D. Appleton & Co., occupies a position among

American medical journals, not unlike that of *The Lancet* in England. It publishes a constant succession of original articles of the most sterling character, its reports of lectures and clinics are always full and instructive, and its editorials are able and interesting. So comprehensive is it that its table of contents covers nearly the whole of one of its broad pages. It may well be believed then, that it contains matter adapted, not alone to the general practitioner, but to every one who is interested in sanitary science. Published weekly at $5.00 per annum.

THE WEEKLY MEDICAL REVIEW, published simultaneously in Chicago and St. Louis, has a corps of editors and contributors that would secure the success of a journal whose home was in less important medical centres than the two cities named. Each section of this great country has diseases peculiar to itself, and the practitioner who would know what progress is made in their treatment must read his home literature, while he who would keep abreast the advance of thought in the whole country needs journals from all sections. No one can fail to know the progress of medical science in the great west, at least, who is a reader of the *Review.* The subscription price is but $3.00 a year.

---

*An Index to the Practice of Medicine;* by WESLEY M. CARPENTER, M. D., Assistant Pathologist to Bellevue Hospital, etc., etc., etc. New York: Wm. Wood & Co., 1883. Price $2.50 and $3.50.

We are indebted to the publishers, and to Mr. J. H. Mattison, No. 11 West Eagle Street, Buffalo, for a copy of this excellent work. It is one of those exceedingly convenient hand-books that every physician should keep upon his table, within easy reach of his hand. There is no particular claim for originality set up in the preparation of this volume, as the material has been mainly obtained from recent medical writers, but it is an excellent compendium of the views of the best authorities. As such it affords to the busy practitioner the latest ideas in symptomatology in a convenient and condensed form.

But it is to the student and to the young practitioner that it will prove of the greatest value. All the principal diseases are considered under the head of symptoms, etiology, physical signs, diagnosis, and treatment, and thus a glance suffices to give the enquirer a fair idea of what is most necessary to know concerning any case which may present itself. Every alternate page is blank, and so opportunity is given for the comparison and record of cases occurring in practice. The binding is flexible morocco with gilt edges, and there is a pocket for memorandum paper, and convenience for carrying a pencil. It has 284 pages, a very complete index, with tables of preparations for sub-cutaneous injections, atomization, weights, measures, etc. We especially commend it to all young men, and to those who are not in full medical practice.

---

*A Treatise on Artificial Crowns; Historical and Descriptive;* by JAMES E. DEXTER, M. D. S. A paper read before the First District Dental Society of the State of New York, January and February, 1883. *Reprint from the Dental Cosmos.*

Within a few years the practice of engrafting artificial crowns upon the roots of decayed teeth has grown to large proportions. Stumps, which formerly were universally condemned to the forceps, are now by means of the many methods offered, readily restored to usefulness and made capable of long years of service. It is at times a matter requiring considerable reflection to correctly decide upon which method will be most useful and permanent in a given case. Dr. Dexter has rendered a service to the profession by presenting a very complete list of them, with an analysis of the peculiar advantages presented by each. He has done even more than this: he has traced their history from the day of the insertion of the old wooden pivot tooth down to the present time, and given a complete resume of the gradual advances made in this department of dentistry.

The pamphlet of thirty-nine pages is illustrated with numerous cuts, which with the descriptions given, are quite sufficient to enable any ingenious dentist to make and insert any of the many forms of artificial crowns.

